# Characterization of immune infiltration in sarcomatoid hepatocellular carcinoma

**DOI:** 10.18632/aging.203076

**Published:** 2021-06-03

**Authors:** Chubin Luo, Haoyang Xin, Dan Yin, Tongyi Zhao, Zhiqiang Hu, Zhengjun Zhou, Rongqi Sun, Na Yao, Qiman Sun, Jia Fan, Xiaowu Huang, Jian Zhou, Shaolai Zhou

**Affiliations:** 1 Department of Liver Surgery and Transplantation, Liver Cancer Institute, Zhongshan Hospital, Fudan University, Shanghai 200032, China; 2 Key Laboratory of Carcinogenesis and Cancer Invasion, Fudan University, Shanghai 200032, China

**Keywords:** sarcomatoid hepatocellular carcinoma, immune infiltration, prognosis

## Abstract

Sarcomatoid hepatocellular carcinoma (sHCC) is a rare type of liver malignancy. Currently, the tumor immune features of sHCC are poorly understood. We recruited 31 patients with resected sHCC for whom tissue samples and complete clinicopathologic and follow-up data were available. To understand the immune infiltration of sHCC, immunohistochemical staining was performed on the resected sHCC samples to compare the expressions of programmed death-1 (PD-1), programmed death-ligand 1 (PD-L1), B7-H3, indoleamine 2,3-dioxygenase (IDO), lymphocyte-activation gene 3 (LAG-3), CD8, FOXP3, and CD68 in tumor and peritumoral tissues. Kaplan-Meier and Cox regression analyses were used to assess the predictive value of immune markers. Sarcomatoid components were characterized with significantly higher expression of PD-L1 and B7-H3 in tumor cells than in conventional HCC components, as well as in peritumoral tissue. Additionally, sarcomatoid components had a higher density of FOXP3^+^ and LAG-3^+^ cells and a lower density of CD8^+^ cells than conventional HCC components or peritumoral tissue. Higher expression of PD-L1 in tumor cells significantly correlated with higher densities of CD8^+^, PD-1^+^, and LAG-3^+^ cells. Increased tumor PD-L1 expression and decreased CD8^+^ T-cell density were associated with poor overall survival (OS) and disease-free survival (DFS) in patients of sHCC. These findings suggest further characterization on relative mechanism of sHCC immune infiltration may identify therapeutic targets for immunotherapy.

## INTRODUCTION

Sarcomatoid hepatocellular carcinoma (sHCC) is a relatively rare malignant liver tumor, accounting for 1.8-2.0% of surgical cases [1, 2]. It is usually diagnosed by a postoperative pathological examination that reveals relative features of spindle or giant cells. Currently, surgical resection is used as the most effective treatment for sHCC [3]. However, the prognosis remains unsatisfactory due to the higher rate of recurrence and metastasis in patients with sHCC than those with non-sHCC [4, 5]. Therefore, new and effective treatment options are required to improve the prognosis of patients with sHCC.

Immune checkpoint proteins, such as PD-1 and PD-L1, protect the host against autoimmunity. However, tumors were also found to co-opt several immune checkpoint proteins to maintain immune escape [6, 7]. Based on the previous findings, immune checkpoint inhibitors were successfully used in the clinical managements of multiple malignancies [8–10]. Currently, immunotherapy is not routinely given to patients with sHCC due to the rare nature of this disease and the uncertainty of surrounding treatment response. Additionally, both immune microenvironment and mechanisms of immune evasion associated with sHCC remain unknown. Thus, analysis on the relative tumor immune microenvironment is important to understand the relationships between patients’ specific tumor types and their immune systems.

To our knowledge, no previous report showed the specific evaluations on the expressions of immune checkpoint proteins, such as PD-L1, B7-H3, IDO, PD-1, and LAG-3, in patients with sHCC. This study assessed the expressions of these immune checkpoint proteins in sHCC samples. Additionally, the cells were evaluated for CD8, FOXP3, and CD68. Our results provide some novel insights into the relative immune microenvironment within sHCC tissues.

## RESULTS

### Typical pathological features of sHCC

**Figure 1:**
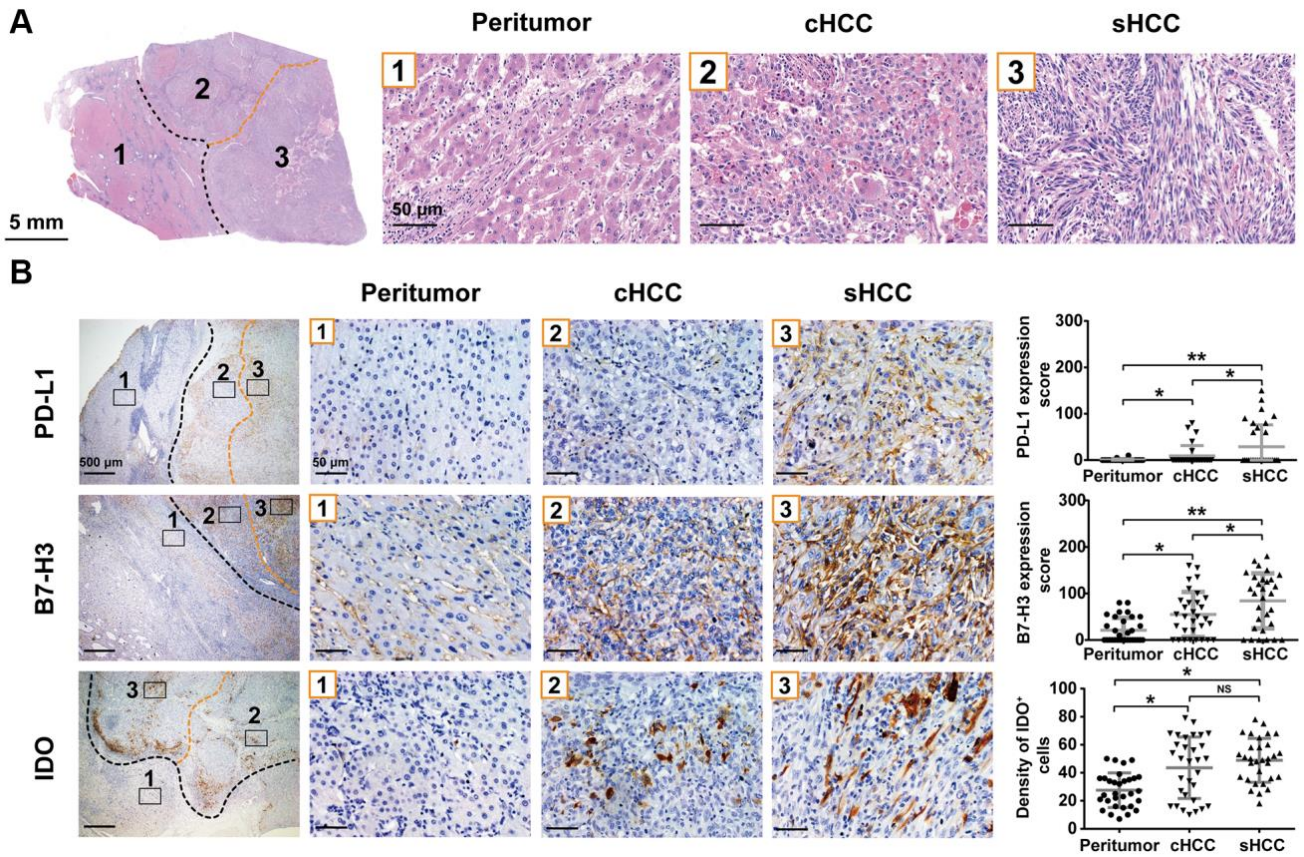
**Typical pathology of sHCC and PD-L1, B7-H3 and IDO expression in the sHCC.** (**A**) Hematoxylin and eosin staining in a sHCC sample. (**B**) Specimens were stained with PD-L1, B7-H3, and IDO, respectively, and the expression in sarcomatoid components, conventional HCC components, and peritumor components are shown. Graph: expression of PD-L1 and B7-H3, as well as IDO^+^ cell densities (cells/mm^2^) of each region are indicated in dot plot. *p< 0.05, **p< 0.01. NS, not significant.

As shown in Figure 1A, sHCC was characterized by pleomorphic spindle cells. Observation indicated that the sarcomatoid components consisted of long and short spindle-shaped cells, marked by elongated nuclei with conspicuous nucleoli and spindle-shaped eosinophilic cytoplasm.

### Expressions of PD-L1, B7-H3, and IDO in sHCC

PD-L1 expression was significantly higher in tumor tissue than in peritumoral tissue (Figure 1B). Of the 31 samples analyzed, 10 (32%) exhibited PD-L1 expression on the tumor cell membrane, whereas only 2 (6.5%) showed PD-L1 expression in the nonneoplastic hepatocytes. In tumor tissue, PD-L1 expression was significantly higher in sarcomatoid components than in conventional HCC components (p < 0.05). Notably, it was difficult to visualize PD-L1 expression in stromal cells under our immunohistochemistry assay (Supplementary Figure 1). Therefore, our study work started to focus on PD-L1 expression in tumor cells only.

The expression of B7-H3, as a member of the B7 family [11], was also evaluated in sHCC. Across all samples analyzed, B7-H3 expression was higher in tumor tissue than in peritumoral tissue. In tumor tissue, sarcomatoid components exhibited significantly higher B7-H3 scores than conventional HCC components (p < 0.05).

Beside B7-H3, another major inhibitor of immune responses is IDO, and its expression in tumors is associated with a worse prognosis [12]. In sHCC tissue, peritumoral tissue exhibited lower IDO^+^ cell density (27.6 ± 2.2 [mean ± standard deviation] cells/mm^2^) than tumor tissue. However, IDO expression was not significantly different between sarcomatoid components and conventional HCC components (48.9 ± 2.8 vs. 43.6 ± 3.9 cells/mm^2^).

### CD8^+^, FOXP3^+^, CD68^+^, PD-1^+^, and LAG-3^+^ cell densities in sHCC

**Figure 2:**
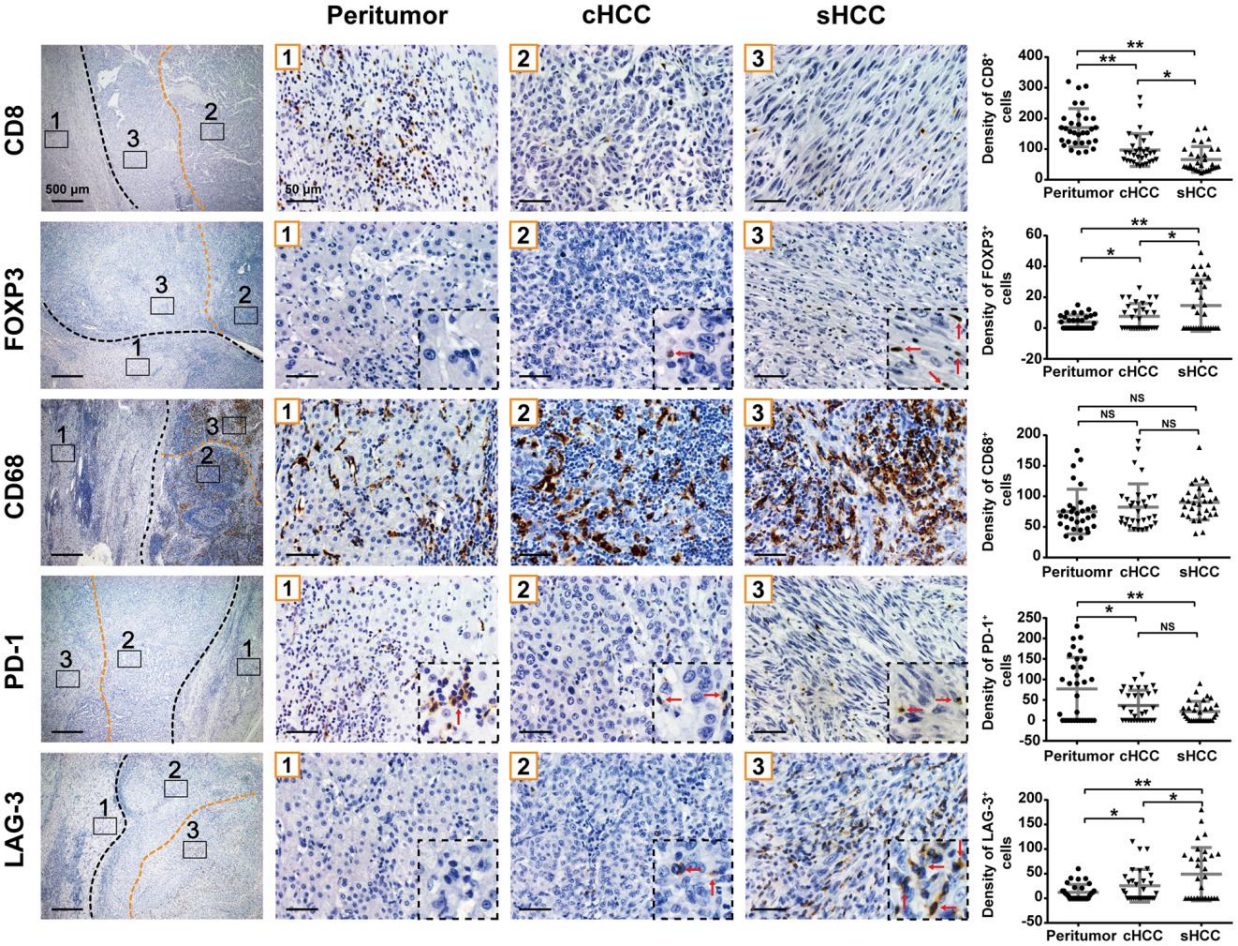
**CD8^+^, FOXP3^+^, CD68^+^, PD-1^+^, and LAG-3^+^ cell densities (cells/mm^2^) in the sarcomatoid components, conventional HCC components and peritumor components are shown.** Graph: cell densities of each region are indicated in dot plot. *p< 0.05, **p< 0.01. NS, not significant.

Because T cells play a critical role in tumor immunity, cytotoxic T cells (CD8^+^) and regulatory T cells (FOXP3^+^) were measured in peritumoral, conventional HCC, and sarcomatoid regions for each sample. The average CD8^+^ cell density was significantly higher in peritumoral tissue (169.8 ± 11.2 cells/mm^2^) than in tumor tissue (Figure 2). Additionally, conventional HCC components exhibited a significantly higher density of CD8^+^ cells than sarcomatoid components (97.0 ± 9.5 vs. 66.3 ± 7.5 cells/mm^2^). The average FOXP3^+^ cell density was lower in peritumoral tissue than in tumor tissue. In tumors, FOXP3^+^ cells had higher density in sarcomatoid components than in conventional HCC components.

Tumor-associated macrophages (TAMs) are key components of immune cells in tumor microenvironment [13–15]. However, no significant difference in density of CD68^+^ TAMs was found among peritumoral tissue, conventional HCC components, and sarcomatoid components (75.1 ± 6.6, 82.5 ± 6.8, and 90.19 ± 5.1 cells/mm^2^, respectively).

PD-1 and LAG-3 are two major immune checkpoint proteins to induce T-cell inactivation. Similar to CD8^+^ T cells, PD-1^+^ lymphocytes were found to have higher concentration in peritumoral tissue (76.8 ± 13.8 cells/mm^2^) than in either sarcomatoid or conventional HCC components. Although the PD-1^+^ cell density was lower in sarcomatoid components than in conventional HCC components (21.7 ± 4.4 vs. 36.5 ± 6.7 cells/mm^2^, respectively), this difference was still not statistically significant. The density of LAG-3^+^ tumor-infiltrating lymphocytes was lower in peritumoral tissue than in tumor tissue. In tumors, the density of LAG-3^+^ tumor-infiltrating lymphocytes was higher in sarcomatoid components than in conventional HCC components.

### Association between CD8^+^ T cells and expressions of immune checkpoint markers in sHCC

**Figure 3:**
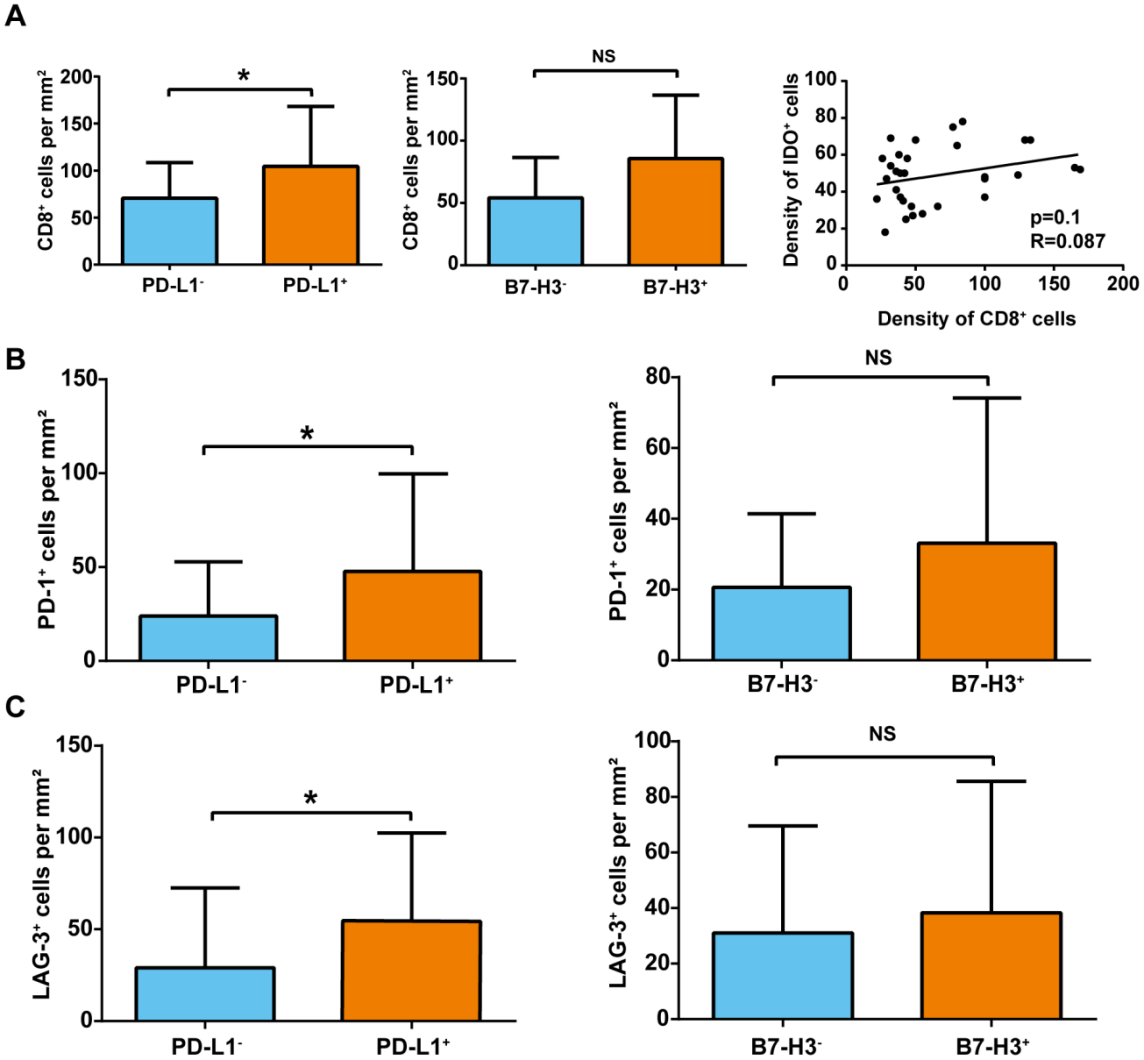
**Association of CD8^+^, PD-1^+^, and LAG-3^+^ cell density and immune-checkpoint marker expression in sHCC.** (**A**) Association of CD8^+^ T-cell density and immune-checkpoint marker expression. *p< 0.05. (**B**) PD-1^+^ cell density (cells/mm^2^) in tumors with and without PD-L1 and B7-3 expression. *p< 0.05. (**C**) LAG-3^+^ cell density (cells/mm^2^) in tumors with and without PD-L1 and B7-3 expression. *p< 0.05. NS, not significant.

Given that PD-L1 and B7-H3 are associated with both T-cell activation and infiltration in tumors, levels of CD8^+^ T-cell densities were compared in PD-L1^+^ sHCC and PD-L1^−^ sHCC samples. As shown in Figure 3A, PD-L1^+^ sHCC samples had a significantly higher CD8^+^ T-cell density than PD-L1^−^ sHCC samples (104.5 ± 14.2 cells/mm^2^ vs. 70.7 ± 5.8 cells/mm^2^, respectively). Additionally, levels of CD8^+^ T-cell densities were also compared in B7-H3^+^ sHCC and B7-H3^−^ sHCC samples. Although B7-H3^+^ sHCC samples had higher CD8^+^ T-cell density than B7-H3^−^ sHCC samples (85.7 ± 6.9 cells/mm^2^ vs. 54.1 ± 11.4 cells/mm^2^, respectively), the difference was still not statistically significant. Furthermore, a positive linear relationship was observed between IDO^+^ cell density and CD8^+^ T-cell density in tumors (R = 0.09); however, the difference was not statistically significant (p = 0.10).

### Relationships among immune checkpoint markers in sHCC

Next, PD-1^+^ and LAG-3^+^ cells were compared in PD-L1^+^ and PD-L1^−^ sHCC samples. As shown in Figure 3B, 3C, PD-1^+^ and LAG-3^+^ cell densities were higher in PD-L1^+^ tumor samples than in PD-L1^−^ tumor samples (PD-1^+^: 47.3 ± 11.7 vs. 24.0 ± 4.5 cells/mm^2^; LAG-3^+^: 54.8 ± 10.7 vs. 29.1 ± 6.7 cells/mm^2^). Additionally, B7-H3^+^ tumor samples exhibited higher PD-1^+^ and LAG-3^+^ cell densities than B7-H3^−^ tumor samples. However, the difference was also not statistically significant (PD-1^+^: 33.09 ± 5.6 vs. 20.6 ± 7.3 cells/mm^2^; LAG-3^+^: 38.3 ± 6.4 vs. 31.0 ± 13.6 cells/mm^2^).

### Prognostic factors

Our studied patient population had 1-, 3-, and 5-year OS rates of 67.7%, 58.0%, and 51.6%, respectively. The 1-, 3-, and 5-year DFS rates were 67.7%, 54.8%, and 40.1%, respectively.

**Figure 4:**
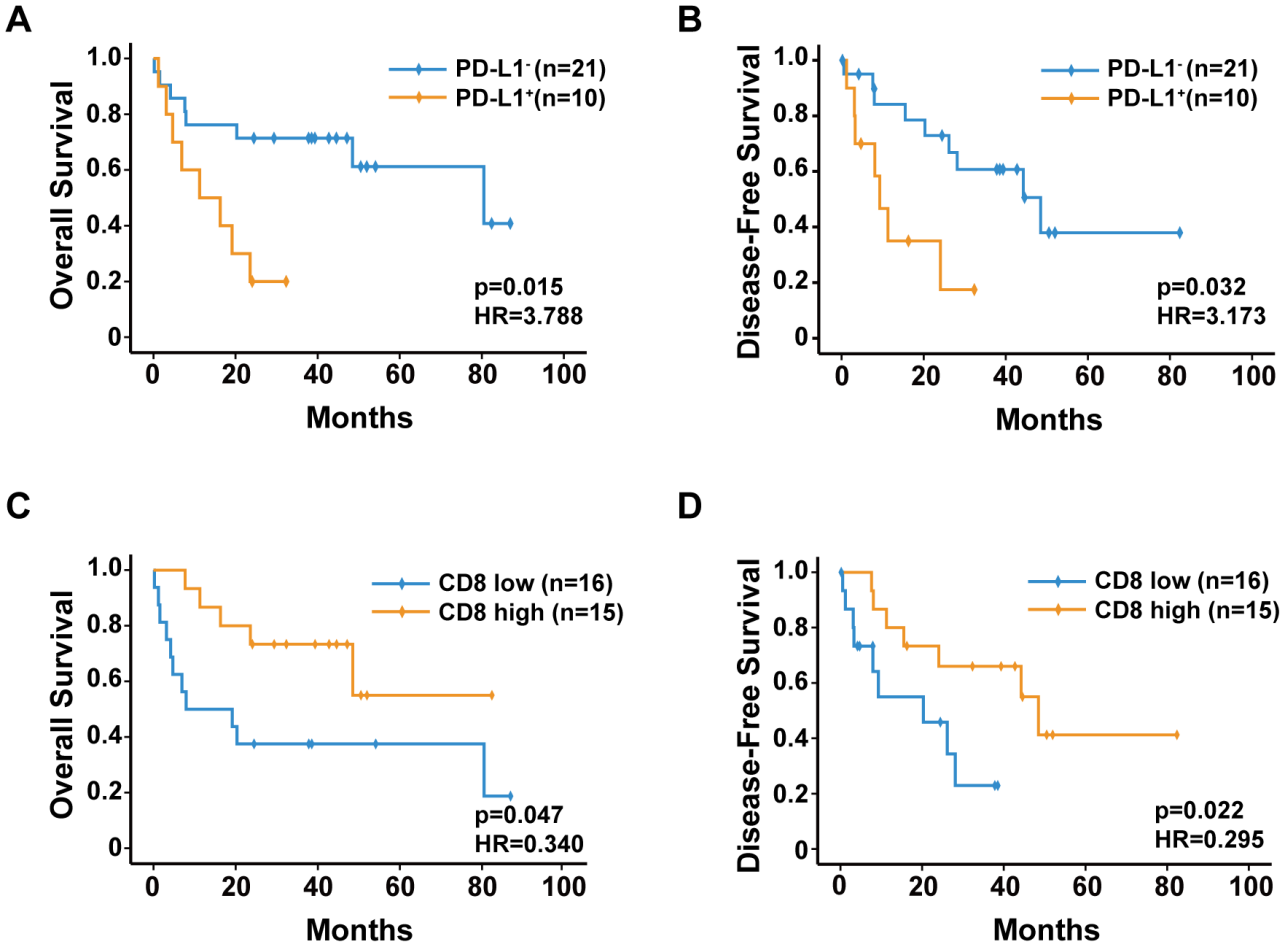
Kaplan-Meier analysis of overall survival and disease-free survival for (**A**, **B**) PD-L1 expression, (**C**, **D**) CD8^+^ T-cell density.

Results of the Kaplan-Meier analysis revealed that 1-, 3-, and 5-year OS rates in sHCC patients with PD-L1^+^ expression (50.0%, 20.0%, and 20.0%, respectively) were significantly lower than those in sHCC patients with PD-L1^−^ expression (76.2%, 71.4%, and 66.7%, respectively) (Figure 4A). Moreover, the 1-, 3-, and 5-year DFS rates in sHCC patients with PD-L1^+^ expression (40.0%, 30.0%, and 30.0%, respectively) were significantly lower than those in sHCC patients with PD-L1^−^ expression (80.9%, 61.9%, and 52.3%, respectively) (Figure 4B). By contrast, the 1-, 3-, and 5-year OS rates in sHCC patients with a high density of CD8^+^ cells (86.7%, 73.3%, and 66.7%, respectively) were significantly higher than those in sHCC patients with a low density of CD8^+^ cells (50.0%, 37.5%, and 37.5%, respectively) (Figure 4C). Additionally, the 1-, 3-, and 5-year DFS rates in sHCC patients with a high density of CD8^+^ cells (80.0%, 60.0%, and 53.3%, respectively) were significantly higher than those in sHCC patients with a low density of CD8^+^ cells (56.3%, 37.5%, and 37.5%, respectively) (Figure 4D).

Results of the univariate analysis indicated that the following factors did not predict both OS and DFS: age, sex, hepatitis B virus, liver cirrhosis, alpha-fetoprotein concentration, alanine aminotransferase concentration, tumor number, and tumor encapsulation. For DFS, the tumor size, PD-L1 expression, and CD8^+^ cell density were the predictors. Similarly, these three predictors, as well as vascular invasion, were associated with OS (Table 1). For the multivariate Cox proportional hazards analyses, the covariates were clinicopathologic features that showed significance in the univariate analysis (Table 2). Vascular invasion remained association with OS (p = 0.028). Additionally, both PD-L1 expression and CD8^+^ cell density were independent prognostic factors for both improved OS (p = 0.012 and p = 0.014, respectively) and DFS (p = 0.016 and p = 0.009, respectively).

**Table 1:** Univariate analyses of factors associated with survival and recurrence.

**Variable**	**OS**				**DFS**		
	**HR**	**95% CI**	**p**		**HR**	**95% CI**	**p**
Age, years (≤51 vs >51)	0.905	0.333 to 2.462	0.845		1.297	0.470 to 3.575	0.616
Sex (female vs male)	1.686	0.221 to 12.84	0.614		2.597	0.338 to 19.941	0.359
HBV (negative vs positive)	0.370	0.048 to 2.832	0.338		1.652	0.374 to 7.308	0.508
AFP, ng/mL (≤20 vs >20)	1.336	0.494 to 3.615	0.568		1.246	0.476 to 3.260	0.654
ALT, U/L (≤40 vs >40)	1.249	0.395 to 3.941	0.705		2.299	0.657 to 8.045	0.193
Liver Cirrhosis (no vs yes)	1.417	0.384 to 5.222	0.601		1.196	0.343 to 4.173	0.779
Tumor size, cm (≤5 vs >5)	4.265	1.194 to 15.230	**0.026**		3.021	1.023 to 8.921	**0.045**
Tumor number (single vs multiple)	1.070	0.386 to 2.968	0.897		0.979	0.353 to 2.715	0.957
Tumor encapsulation (complete vs none)	2.211	0.709 to 6.897	0.171		1.667	0.608 to 4.570	0.312
Vascular invasion (no vs yes)	4.291	1.198 to 15.362	**0.025**		1.316	0.500 to 3.468	0.578
PD-L1 (negative vs positive)	3.788	1.298 to 11.054	**0.015**		3.173	1.106 to 9.101	**0.032**
B7-H3 (negative vs positive)	1.298	0.289 to 5.750	0.739		1.728	0.393 to 7.590	0.469
IDO (low vs high)	1.278	0.454 to 3.593	0.641		1.291	0.493 to 3.376	0.603
CD8 (low vs high)	0.340	0.117 to 0.988	**0.047**		0.295	0.100 to 0.871	**0.027**
FOXP3 (low vs high)	1.744	0.648 to	0.805		1.133	0.433 to 2.963	0.799
CD68 (low vs high)	0.881	0.325 to 2.376	0.803		1.655	0.623 to 4.396	0.312
PD-1 (low vs high)	0.907	0.339 to 2.432	0.847		1.464	0.556 to 3.858	0.441
LAG-3 (low vs high)	1.378	0.483 to 3.936	0.549		1.485	0.551 to 4.001	0.434

Note: Cox proportional hazards regression model.

The immune markers were analyzed across the whole tumor tissues.

Abbreviations: HBV, hepatitis B virus; AFP, alpha-fetoprotein; ALT, alanine aminotransferase; OS, overall survival; DFS, disease-free survival; HR, hazard ratio; CI, confidential interval.

**Table 2:** Multivariate analyses of factors associated with survival and recurrence.

**Survival**	**HR**	**95% CI**	**p**
OS Tumor size, cm (≤5 vs >5)	3.647	0.832 to 15.997	0.748
Vascular invasion (no vs yes)	4.931	1.190 to 20.430	**0.028**
PD-L1 (negative vs positive)	5.696	1.473 to 22.018	**0.012**
CD8 (low vs high) DFS	0.178	0.044 to 0.722	**0.016**
Tumor size, cm (≤5 vs >5)	1.613	0.508 to 5.120	0.418
PD-L1 (negative vs positive)	5.036	1.382 to 18.354	**0.014**
CD8 (low vs high)	0.186	0.053 to 0.651	**0.009**

Note: Cox proportional hazards regression model.

The immune markers were analyzed across the whole tumor tissues.

Abbreviations: OS, overall survival; DFS, disease-free survival; HR, hazard ratio; CI, confidential interval.

## DISCUSSION

As a rare histological subtype of HCC for sarcomatoid, both etiology and pathogenesis have not yet been fully elucidated. Novel therapeutic strategies are still expected to improve the survival rates in patients with sHCC. Recently, immunotherapy has emerged as a new and promising approach in tumor treatments. In order to potentially enhance the relative therapy development, the details of immune infiltration in sHCC must be precisely characterized.

For this aim, our present study assessed the expressions of several immune checkpoint markers in different sHCC components, including PD-L1, B7-H3, IDO, PD-1, and LAG-3. Additionally, CD8^+^, FOXP3^+^, and CD68^+^ immune cells were also evaluated. PD-L1 expression was found on the tumor cell membrane in 32% of sHCC patients, and was significantly higher in sarcomatoid components than in conventional HCC components. Sarcomatoid components in tumor tissue showed an increased level of immunosuppressive activities in sarcomatoid microenvironment, likely accounting for the progression of this type of tumor. Tumor samples of patients with PD-L1^+^ cells exhibited the increased levels of both PD-1 and LAG-3 expressions in tumor-infiltrating lymphocytes. Additionally, tumors with PD-L1^+^ cells also had significantly higher density of CD8^+^ T cells than those without PD-L1 expression cells, suggesting that a mechanism of adaptive immune resistance may be active in sHCC and may also be overcome by anti-PD-1/PD-L1 administration. Results of Kaplan-Meier analyses showed that both increased tumor PD-L1 expression and decreased CD8^+^ T-cell density were associated with the worse values of both OS and DFS in patients with sHCC.

Belonging to the B7 family of immune regulatory ligands, B7-H3 acts as a co-signaling transmembrane protein. It has the increased expression in various tumor cells, including tumors of the pancreas, gastrointestinal tract, breast, ovaries, kidneys, lungs, and head and neck, and is correlated with a worse prognosis [16–22]. Even though it is still uncertain whether B7-H3 plays a direct role in inhibiting T-cell function [23], our study revealed majority of sHCC patients had B7-H3-expressing tumor cells in both sarcomatoid and conventional HCC components. Tumors with B7-H3 expression also had the increased expression levels of both PD-1 and LAG-3 in tumor-infiltrating lymphocytes, and had higher density of CD8^+^ T cells than those without B7-H3 expression. However, these found differences were not statistically significant, probably because of limitation in sample amount. Thus, future study will be planned to make up this analysis and to comprehensively evaluate the role of B7-H3 in the tumor microenvironment of sHCC.

Previous studies revealed that sHCC was characterized by the epithelial-mesenchymal transition (EMT), which carries the crucial functions in tumor immunosuppression and immune evasion [24–26]. For example, as a major activator of the EMT, transforming growth factor beta upregulates PD-L1 expression through the phosphoinositide 3-kinase/AKT [27]. Snail, another major activator of EMT, upregulates CXC chemokine ligand 2 expression via nuclear factor kappa B signaling to increase myeloid-derived suppressor cells infiltration, leading to CD8^+^ T-cell inhibition and tumor progression [28]. Thus, we hypothesize that the EMT may involve into the immunosuppression in sHCC. Future investigations will be aimed to uncover the relative underlying mechanism of immunosuppression in sHCC.

Our present study has two key limitations. First, it was a single-center study with the limited numbers of sHCC patients. However, given the rare nature of sHCC for difficulty to obtain the clinical cases, the present cohort should be regarded as relatively large. Second, the multiple color immunofluorescence assay was not conducted in study to analyze the co-expressions of immune-specific markers due to the lack of multiplex capability in our analytical tools.

The establishments of effective therapies are urgently expected to treat patients of sHCC. The results of this study support the rationale to target the immune checkpoint axes in sHCC. The increased levels of PD-L1, B7-H3, and IDO expressions in sHCC cells may play an immunosuppressive role in tumor microenvironment, suggesting that co-targeting PD-L1 along with other immune checkpoints may be a potential strategy to treat patients with sHCC. Recently, PD-1 inhibition combining with either B7-H3 or IDO inhibitions have already been tested in clinical trials for some solid tumors [29, 30]. Given the present findings of upregulations of multiple immune checkpoints in our cohort, future investigation will be to test the therapeutic potential of targeting immune checkpoint proteins as a novel approach to treat patients of sHCC.

## MATERIALS AND METHODS

### Patient information and follow-up details

Between March 2013 and April 2017, 53 sHCC tissue samples were collected from patients undergoing surgical resection at the Zhongshan Hospital, Fudan University (Shanghai, China). Patients with unavailable tumor and peritumoral samples or incomplete clinicopathologic and follow-up data were excluded from the study. In total, our study included 31 patients with sHCC. The clinical information of these patients is listed in the Supplementary Table 1.

Hematoxylin and eosin staining and immunohistochemical staining (Hep Par-1, arginase-1, cytokeratin, and vimentin) were reviewed by two pathologists to confirm the pathological diagnosis of sHCC. All tissue samples from 31 sHCC patients were mixed with peritumoral, conventional HCC, and sHCC components. All patients gave their written informed consent for their samples to be used in our study. Surveillance of postsurgical patients was described in a previous study [31].

### Immunohistochemical staining assay and evaluation

Immunohistochemical staining was conducted on whole-slide serial tissue sections from FFPE surgical resection specimens to determine the expression levels for PD-L1, B7-H3, IDO, CD8, FOXP3, CD68, PD-1, and LAG-3, according to standardized institutional protocols as previously described [32]. The reagents used for this evaluation are listed in Supplementary Table 2.

Briefly, one representative FFPE block from each tumor specimen (n = 31) was selected with tumor and peritumoral liver tissue present on the same block. Consecutive 5-μm-thick sections were sliced from each block and placed on cleaned glass slides. After deparaffinization/rehydration, FPEE sections were subjected to endogenous peroxidase blocking, heat-induced antigen retrieval, antibody incubation at specific dilutions, and signal detection.

Two pathologists who were blinded to the clinical outcomes independently assessed the immunostaining results. A cell membrane staining algorithm was applied to analyze the levels of PD-L1 and B7-H3 expression in tumor cells. This expression was quantified using a four-value staining intensity score (0, none; 1, weak; 2, moderate; 3, strong) and the extension (percentage) of expression. H-score was calculated by multiplying the intensity and reactivity extension values (H-score range, 0–300) [33]. For both PD-L1 and B7-H3, tumor cells with expression levels greater than 5% were considered to be positive. For the profiling analysis of CD8, FOXP3, CD68, PD-1, LAG-3, and IDO, cell density was measured by counting the number of positive cells expressing these markers in three randomly selected fields. For each marker, the average of these values was expressed as the number of cells per square millimeters.

### Statistical analyses

The mean expression levels of PD-L1, B7-H3, IDO, CD8, FOXP3, CD68, LAG-3, and PD-1 in various tissue compartments (peritumoral, conventional HCC component, and sarcomatoid component) were compared using Wilcoxon signed-rank tests. The statistical analyses were conducted using STATA 14.0 (Stata Corp.). A correlation analysis was performed using Spearman linear correlation analysis. The Kaplan-Meier method was used to determine OS and DFS, and the log-rank test was applied to analyze the differences. Univariate and multivariate analyses were conducted using the Cox proportional hazards regression model. A p-value less than 0.05 was statistically significant.

## Supplementary Material

Supplementary Figure 1

Supplementary Tables
