# Supplementary figures and images for: Characterization of immune infiltration in sarcomatoid hepatocellular carcinoma
Source: Aging (Albany NY). 2021 Jun 3;13(11):15126–38. doi: 10.18632/aging.203076 (PMC8221324; doi:10.18632/aging.203076)

## SUPPLEMENTARY FIGURE

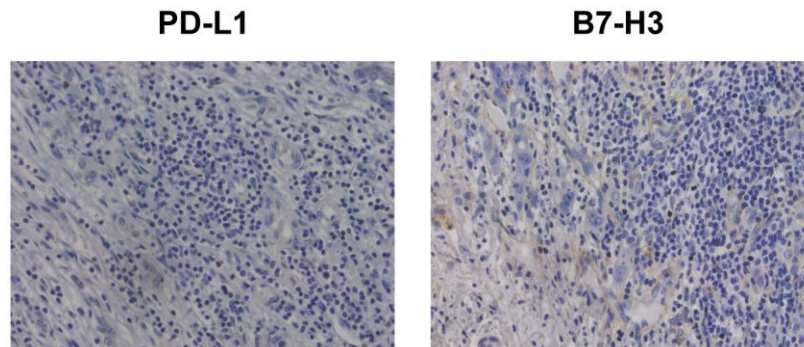

Supplementary Figure 1. PD-L1 and B7-H3 expression in the stromal cells.

Supplement: Supplementary Figure 1 [file aging-13-203076-s001.pdf]
