# Supplementary material for: Characterization of immune infiltration in sarcomatoid hepatocellular carcinoma
Source: Aging (Albany NY). 2021 Jun 3;13(11):15126–38. doi: 10.18632/aging.203076 (PMC8221324; doi:10.18632/aging.203076)
Supplement: Supplementary Tables [file aging-13-203076-s002.pdf]

SUPPLEMENTARY TABLES

Supplementary Table 1. Patient and tumor characteristics.

Clinicopathologic characteristics	N (%)
Age, years	
≤51	19 (61.3)
>51	12 (38.7)
Sex	
female	3 (9.7)
male	28 (90.3)
HBV	
negative	4 (12.9)
positive	27 (87.1)
AFP, ng/mL	
≤20	15 (48.4)
>20	16 (51.6)
ALT, U/L	
≤40	10 (32.3)
>40	21 (67.7)
Liver cirrhosis	
no	6 (19.4)
yes	25 (80.6)
Tumor size, cm	
≤5	13 (41.9)
>5	18 (58.1)
Tumor number	
single	18 (58.1)
multiple	13 (41.9)
Tumor encapsulation	
complete	18 (58.1)
none	13 (41.9)
Vascular invasion	
no	11 (35.5)
yes	20 (64.5)

Abbreviations: HBV, hepatitis B virus; AFP, alpha-fetoprotein; ALT, Alanine aminotransferase.

Supplementary Table 2. Immunohistochemical staining procedures.

Antibody	Clone	Source	Antigen retrieval	Primary Ab incubation
PD-L1	E1L3N	CST	Citrate buffer, pH 6.0	Overnight, 4° C
B7-H3	D9M2L	CST	Citrate buffer, pH 6.0	Overnight, 4° C
IDO	D5J4E	CST	Citrate buffer, pH 6.0	Overnight, 4° C
CD8	C8/144B	CST	Citrate buffer, pH 6.0	Overnight, 4° C
FOXP3	D2W8E	CST	Citrate buffer, pH 6.0	Overnight, 4° C
CD68	D4B9C	CST	Citrate buffer, pH 6.0	Overnight, 4° C
PD-1	EH33	CST	Citrate buffer, pH 6.0	Overnight, 4° C
LAG-3	D2G4O	CST	Citrate buffer, pH 6.0	Overnight, 4° C
